# Crystal structure and Hirshfeld surface analysis of *rac*-2-[2-(4-chloro­phen­yl)-3,4-di­hydro-2*H*-1-benzo­pyran-4-yl­idene]hydrazine-1-carbo­thio­amide

**DOI:** 10.1107/S2056989019005073

**Published:** 2019-04-25

**Authors:** Ruokuosenuo Zatsu, Prabhakar Maddela, M. Indira Devi, Ranjit Singh, Chullikkattil P. Pradeep

**Affiliations:** a Department of Chemistry, Nagaland University, Hqtrs: Lumami, Nagaland-798627, India; b School of Basic Sciences, Indian Institute of Technology Mandi, Mandi-175005, Himachal Pradesh, India

**Keywords:** crystal structure, flavanone, chromane, thio­semicarbazide, Schiff base, N—H⋯S hydrogen bonds, supra­molecular chemistry, Hirshfeld surface analysis

## Abstract

In the title compound, C_16_H_14_N_3_OSCl, a Schiff base derivative of a thio­semicarbazide with a flavanone, the 4-chlorophenyl ring is inclined to the benzene ring of the chromane ring system by 30.72 (12)°. The pyran ring has an envelope conformation with the methine C atom as the flap. The mean plane of the thio­urea unit is twisted with respect to the benzene ring of the chromanone ring system, subtending a dihedral angle of 19.78 (19)°. In the crystal, mol­ecules are linked by two pairs of N—H⋯S hydrogen bonds, forming inversion dimers enclosing *R* ^2^_2_(8) ring motifs, which are linked to form ribbons propagating along the *b*-axis direction. The inter­molecular contacts in the crystal have been analysed using Hirshfeld surface analysis.

## Abstract 2

The title compound, is a Schiff base derivative of a thio­semicarbazide with a flavanone. In the crystal, mol­ecules are linked by two pairs of N—H⋯S hydrogen bonds, forming inversion dimers enclosing 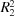
(8) ring motifs, which are linked to form ribbons propagating along the *b*-axis direction.

## Chemical context   

Flavanones, a subclass of flavonoids, are widely recognized for their nutraceutical values (Testai & Calderone, 2017 ▸). Flavanones are also known for their potential bioactivities against cancer (Bauvois *et al.*, 2003 ▸). Thio­semicarbazides are a class of versatile ligands exhibiting important physicochemical properties due to their π-delocalization and flexibility of coordination modes. Therefore, a combination of flavanones and thio­semicarbazides may lead to compounds having synergistic properties of both classes of compounds. Schiff base derivatives of thio­semicarbazides have been studied for their biological and pharmacological properties (Bai *et al.*, 2017 ▸). However, Schiff base derivatives of flavanones with thio­semicarbazides have not been explored extensively (Brodowska *et al.*, 2016 ▸; Bargujar *et al.*, 2018 ▸). In particular, structurally characterized flavanone–thio­semicarbazone Schiff bases are rare in the literature. The presence of NH and S moieties in such compounds opens up the possibility of studying the role of the comparatively less explored class of N—H⋯S inter­actions in building supra­molecular architectures. This is of inter­est as hydrogen bonding to sulfur is known to play an important role in biological systems (Andersen *et al.*, 2014 ▸; Walters *et al.*, 2005 ▸). Considering the above, we have synthesized the title compound through a Schiff base condensation reaction, and report herein on its crystal structure and the Hirshfeld surface analysis.
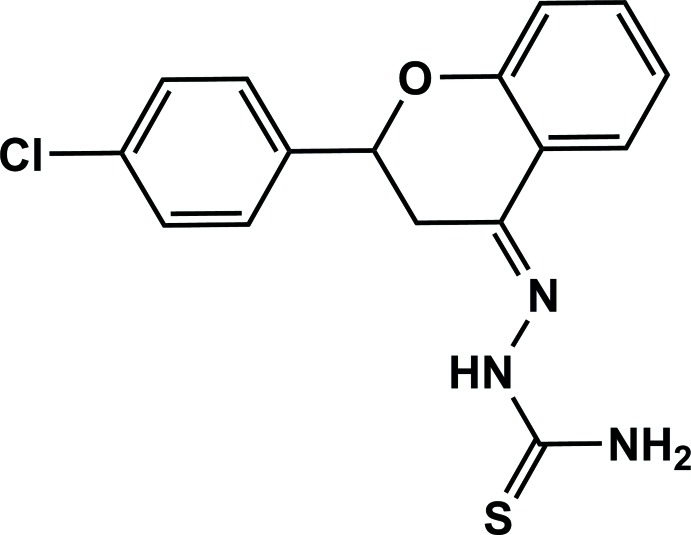


## Structural commentary   

**Figure 1:**
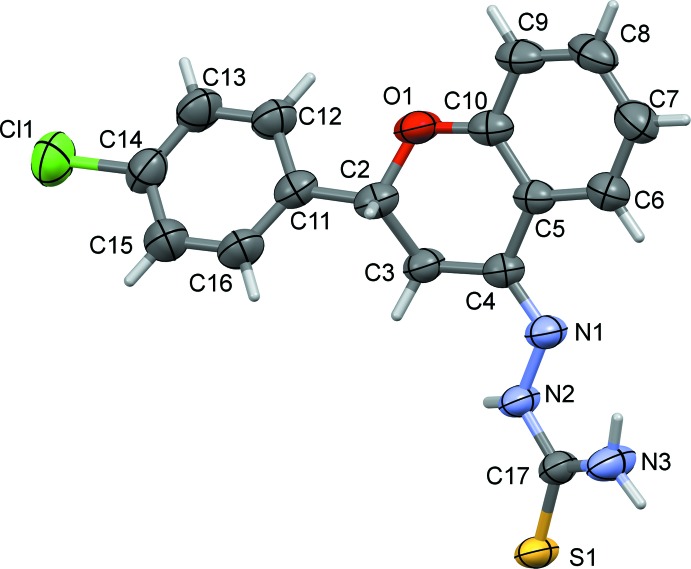
A view of the mol­ecular structure of the title compound, with the atom labelling. Displacement ellipsoids are drawn at the 50% probability level. The orientation of the fiigure means that one of the two H atoms on C3 is not shown.

The mol­ecular structure of the title compound is illustrated in Fig. 1 ▸. The 4-chloro­phenyl ring (C11–C16) is inclined to the benzene ring (C5–C10) of the chromanone ring system by 30.72 (12)°. The pyran ring (O1/C2–C5/C10) has an envelope conformation with atom C2 as the flap, being displaced by 0.655 (2) Å from the mean plane through the other five atoms of the ring. The mean plane of the thio­urea unit (N2/C17/S1/N3) is twisted with respect to benzene ring (C5-C10) of the chromane ring system, forming a dihedral angle of 19.78 (19)°.

## Supra­molecular features   

**Figure 2:**
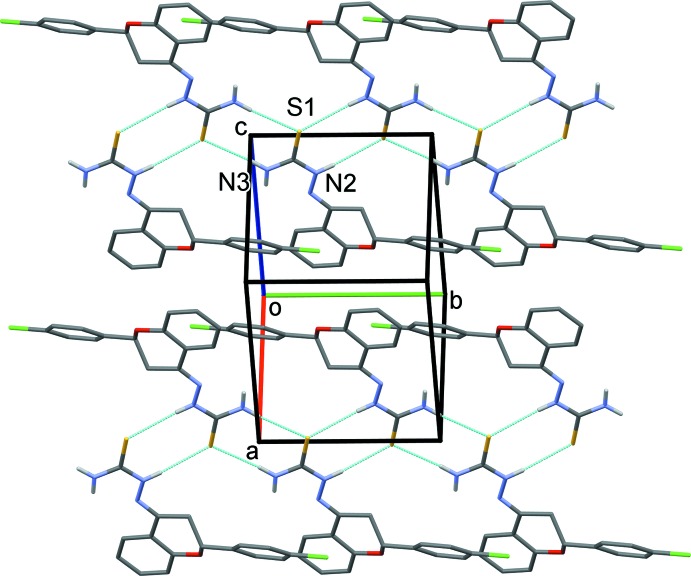
A view normal to plane (101) of the crystal packing of the title compound. The N—H⋯S hydrogen bonds are shown as dashed lines (Table 1 ▸). For clarity, C-bound H atoms have been omitted.

A strong hydrogen bond often involves highly electronegative second row elements such as N, O and F. However, the less electronegative third row elements (P, S and Cl) are also known to take part in hydrogen-bonding inter­actions. In the crystal of the title compound, mol­ecules are linked by two pairs of N—H⋯S hydrogen bonds, forming inversion dimers enclosing *R* ^2^_2_(8) ring motifs, which are linked to form ribbons propagating along the *b*-axis direction (Table 1 ▸ and Fig. 2 ▸). In the crystal, there are no other significant short inter­molecular inter­actions present.

## Hirshfeld surface analysis and two-dimensional fingerprint plots for the title compound   

The Hirshfeld surface analysis (Spackman & Jayatilaka, 2009 ▸) and the associated two-dimensional fingerprint plots (McKinnon *et al.*, 2007 ▸) were performed with *CrystalExplorer17* (Turner *et al.*, 2017 ▸). A recent article by Tiekink and collaborators (Tan *et al.*, 2019 ▸) ‘outlines the various procedures and what can be learned by using *CrystalExplorer*’.

**Figure 3:**
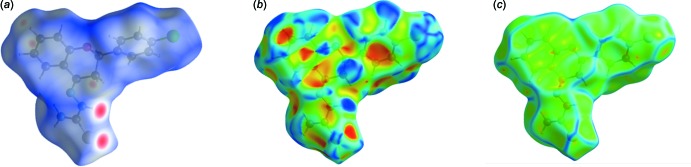
The Hirshfeld surface of the title compound mapped over (*a*) *d*_norm_, −0.3525 to 1.4929 arbitrary units, (*b*) shape-index and (*c*) curvedness.

The Hirshfeld surface of the title compound mapped over *d*_norm_ is given in Fig. 3 ▸ *a*. The red spots indicate specific points of contact in the crystal. The Hirshfeld surface mapped over the shape-index is given in Fig. 3 ▸ *b*, showing red spots and blue regions indicative of possible C⋯H/H⋯C (*i.e*. C—H⋯π) contacts. The Hirshfeld surface mapped over the curvedness is given in Fig. 3 ▸ *c*. Here the region around the chromane ring system is fairly flat, indicative of possible π–π inter­actions. However, these inter­actions must be extremely weak as analysis of the structure using *PLATON* (Spek, 2009 ▸) did not indicate the presence of any significant C—H⋯π or offset π–π inter­actions in the crystal.

**Figure 4:**
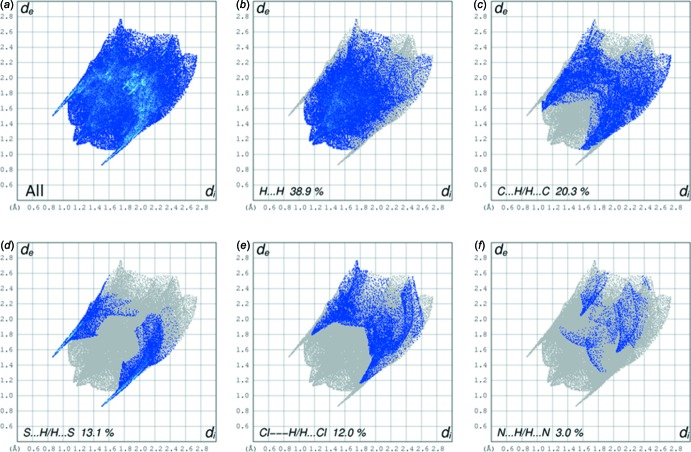
(*a*) The full two-dimensional fingerprint plot for the title compound and fingerprint plots delineated into (*b*) H⋯H, (*c*) C⋯H/H⋯C, (*d*) S⋯H/H⋯S, (*e*) Cl⋯H/H⋯Cl and (*f*) N⋯H/H⋯N contacts.

The full two-dimensional fingerprint plot for the title compound is given in Fig. 4 ▸ *a*. The principal inter­molecular inter­actions (Fig. 4 ▸ *b*–4*f*) are delineated into H⋯H (38.9%), C⋯H/H⋯C (20.3%), S⋯H/H⋯S (13.1%), Cl⋯H/H⋯Cl (12.0%) and N⋯H/H⋯N (3.0%) contacts. Note that only for the H⋯H, C⋯H/H⋯C and S⋯H/H⋯S contacts is *d*_e_ + *d*_i_ (where *d*_e_ and *d*_i_ are the distances from a given point on the surface to the nearest atom outside and inside, respectively), less than the sum of the van der Waals radii of the individual atoms.

## Database survey   

A search of the Cambridge Structural Database (CSD, Version 5.40, update February 2019; Groom *et al.*, 2016 ▸) for a similar structure gave one hit, the compound 2′[(2-(4-fluoro­phen­yl)chroman-4-yl­idene]isonicotinohydrazide (CSD refcode TEJQUV; Nie *et al.*, 2006 ▸). Here, the pyran ring has an envelope conformation and the 4-fluoro­phenyl ring is inclined to the benzene ring of the chromane ring system by 66.57 (11)°. In the title compound, the pyran ring also has an envelope conformation and the 4-chloro­pheny ring is inclined to the benzene ring of the chromane ring system by only 30.72 (12)°.

A search for the 2-(tetra­hydro-4*H*-pyran-4-yl­idene)hydrazine-1-carbo­thio­amide skeleton gave one hit, *viz*. (*E*)-2-[2,6-bis­(4-chloro­phen­yl)-3,5-di­methyl­tetra­hydro-4*H*-pyran-4-yl­idene]hydrazinecarbo­thio­amide (UQAWAL; Umamatheswari *et al.*, 2011 ▸). Here, the pyran ring has a chair conformation and the bond lengths and angles of the hydrazinecarbo­thio­amide unit are similar to those in the title compound.

## Synthesis and crystallization   

The synthesis of the title compound was achieved by following a reported procedure with some modifications (Bargale *et al.*, 1988 ▸). Conc. H_2_SO_4_ (10 mol %) in ethanol (5 ml) was added to a stirred solution of 2-(4-chloro­phen­yl)-chroman-4-one (0.258 g, 1 mmol) (Zheng *et al.*, 2013 ▸) and thio­semicarbazide (0.091 g, 1 mmol). The mixture was refluxed for 96 h with continuous stirring. After completion of the reaction, as monitored by TLC, the solvent was removed under reduce pressure and then ice-cold water was added. The resulting solid product was collected by filtration, washed with water (3–4 times) and finally with hexane and then dried at room temperature. Pale-yellow plate-like crystals of the title compound were obtained by slow evaporation at room temperature of a solution in aceto­nitrile (yield 90%, m.p. 483-486 K). IR (KBr, cm^−1^): 3417, 3245, 3152, 2984, 2888, 2790, 1598, 1512, 1454, 1298, 1250, 1089, 1077, 883, 766, 507, 498. ^1^H NMR (400 MHz, DMSO-*d_6_*), δ ppm: 10.47 (*s*, 1H, NH), 8.32 (*d*, 2H, *J* = 6.50 Hz, NH_2_); 8.13 (*s*, 1H, Ar-H); 7.54 (*dd*, 4H, *J* = 8.41 Hz, Ar-H); 7.35–7.31 (*m*, 1H, Ar-H); 7.02–6.97 (*m*, 2H, Ar-H); 5.25 (*dd*, 1H, *J* = 2.36, 2.40 Hz, CH); 2.79 (*dd*, 1H, *J* = 12.10, 12.0 Hz, CH_2_); 2.51 (*s*, 1H, CH_2_).^13^C NMR (300 MHz, DMSO-*d_6_*), δ ppm: 178.84; 156.71; 141.71; 138.79; 132.76; 131.24; 128.44; 128.27; 125.49; 121.48; 120.10; 117.47; 75.41; 31.83. Analysis calculated for C_16_H_14_N_3_OSCl: C, 57.91; H, 4.25; N, 12.66; S, 9.66. Found: C, 57.85; H, 4.28; N, 12.61; S, 9.59.

## Refinement   

Crystal data, data collection and structure refinement details are summarized in Table 2 ▸. The NH and NH_2_ H atoms were located in a difference-Fourier map and refined freely. The C-bound H atoms were included in calculated positions and treated as riding atoms: C—H = 0.93–0.98 Å with *U*_iso_(H) = 1.2*U*_eq_(C).

## Supplementary Material

Crystal structure: contains datablock(s) I, global. DOI: 10.1107/S2056989019005073/zp2036sup1.cif

Supporting information file. DOI: 10.1107/S2056989019005073/zp2036Isup3.cdx

Structure factors: contains datablock(s) I. DOI: 10.1107/S2056989019005073/zp2036Isup4.hkl

Supporting information file. DOI: 10.1107/S2056989019005073/zp2036Isup4.cml

CCDC reference: 1893434

Additional supporting information:  crystallographic information; 3D view; checkCIF report

## Figures and Tables

**Table 1:** Hydrogen-bond geometry (Å, °)

*D*—H⋯*A*	*D*—H	H⋯*A*	*D*⋯*A*	*D*—H⋯*A*
N2—H2*N*⋯S1^i^	0.85 (3)	2.65 (3)	3.480 (2)	167 (2)
N3—H3*BN*⋯S1^ii^	0.88 (3)	2.52 (3)	3.392 (2)	171 (2)

Symmetry codes: (i) 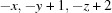; (ii) 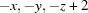.

**Table 2:** Experimental details

Crystal data	
Chemical formula	C_16_H_14_ClN_3_OS
*M*_r_	331.81
Crystal system, space group	Triclinic, *P* 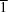
Temperature (K)	293
*a*, *b*, *c* (Å)	7.8218 (7), 8.4207 (6), 12.3402 (11)
α, β, γ (°)	99.838 (7), 95.771 (7), 96.515 (7)
*V* (Å^3^)	789.66 (12)
*Z*	2
Radiation type	Cu *K*α
μ (mm^−1^)	3.41
Crystal size (mm)	0.50 × 0.17 × 0.10

Data collection	
Diffractometer	Rigaku OD, SuperNova, Dual, Cu at zero, Eos
Absorption correction	Gaussian (*CrysAlis PRO*; Rigaku OD, 2015 ▸)
*T*_min_, *T*_max_	0.464, 1.000
No. of measured, independent and observed [*I* > 2σ(*I*)] reflections	4478, 2766, 2346
*R*_int_	0.019
(sin θ/λ)_max_ (Å^−1^)	0.596

Refinement	
*R*[*F* ^2^ > 2σ(*F* ^2^)], *wR*(*F* ^2^), *S*	0.042, 0.121, 1.05
No. of reflections	2766
No. of parameters	211
H-atom treatment	H atoms treated by a mixture of independent and constrained refinement
Δρ_max_, Δρ_min_ (e Å^−3^)	0.51, −0.41

Computer programs: *CrysAlis PRO* (Rigaku OD, 2015 ▸), *SHELXT* (Sheldrick, 2015*a* ▸), *SHELXL2018/03* (Sheldrick, 2015*b* ▸), *OLEX2* (Dolomanov *et al.*, 2009 ▸), *Mercury* (Macrae *et al.*, 2008 ▸), *SHELXL2018/03* (Sheldrick, 2015*b* ▸), *PLATON* (Spek, 2009 ▸) and *publCIF* (Westrip, 2010 ▸).

## supplementary crystallographic information

### Crystal data

**Table:**

C_16_H_14_ClN_3_OS	*Z* = 2
*M**_r_* = 331.81	*F*(000) = 344
Triclinic, *P*1	*D*_x_ = 1.395 Mg m^−^^3^
*a* = 7.8218 (7) Å	Cu *K*α radiation, λ = 1.54184 Å
*b* = 8.4207 (6) Å	Cell parameters from 2014 reflections
*c* = 12.3402 (11) Å	θ = 3.7–66.5°
α = 99.838 (7)°	µ = 3.41 mm^−^^1^
β = 95.771 (7)°	*T* = 293 K
γ = 96.515 (7)°	Plate, yellow
*V* = 789.66 (12) Å^3^	0.50 × 0.17 × 0.10 mm

### Data collection

**Table:**

Rigaku OD, SuperNova, Dual, Cu at zero, Eos diffractometer	2346 reflections with *I* > 2σ(*I*)
Radiation source: micro-focus sealed X-ray tube	*R*_int_ = 0.019
ω scans	θ_max_ = 66.7°, θ_min_ = 3.7°
Absorption correction: gaussian (CrysAlis PRO; Rigaku OD, 2015)	*h* = −9→9
*T*_min_ = 0.464, *T*_max_ = 1.000	*k* = −10→7
4478 measured reflections	*l* = −14→14
2766 independent reflections	

### Refinement

**Table:**

Refinement on *F*^2^	Primary atom site location: structure-invariant direct methods
Least-squares matrix: full	Secondary atom site location: difference Fourier map
*R*[*F*^2^ > 2σ(*F*^2^)] = 0.042	Hydrogen site location: mixed
*wR*(*F*^2^) = 0.121	H atoms treated by a mixture of independent and constrained refinement
*S* = 1.05	*w* = 1/[σ^2^(*F*_o_^2^) + (0.0655*P*)^2^ + 0.1825*P*] where *P* = (*F*_o_^2^ + 2*F*_c_^2^)/3
2766 reflections	(Δ/σ)_max_ = 0.001
211 parameters	Δρ_max_ = 0.51 e Å^−^^3^
0 restraints	Δρ_min_ = −0.41 e Å^−^^3^

### Special details

**Table:**

Geometry. All esds (except the esd in the dihedral angle between two l.s. planes) are estimated using the full covariance matrix. The cell esds are taken into account individually in the estimation of esds in distances, angles and torsion angles; correlations between esds in cell parameters are only used when they are defined by crystal symmetry. An approximate (isotropic) treatment of cell esds is used for estimating esds involving l.s. planes.

### Fractional atomic coordinates and isotropic or equivalent isotropic displacement parameters (Å^2^)

**Table:**

	*x*	*y*	*z*	*U*_iso_*/*U*_eq_	
S1	0.03040 (9)	0.27189 (6)	1.04983 (4)	0.0560 (2)	
Cl1	0.63580 (11)	1.37767 (8)	0.85742 (7)	0.0909 (3)	
O1	0.2996 (2)	0.65276 (19)	0.57103 (12)	0.0597 (4)	
N1	0.1205 (2)	0.3125 (2)	0.75055 (14)	0.0492 (4)	
N2	0.1066 (3)	0.3537 (2)	0.86177 (14)	0.0492 (4)	
H2N	0.089 (3)	0.448 (3)	0.892 (2)	0.061 (7)*	
N3	0.0872 (4)	0.0835 (2)	0.86608 (18)	0.0689 (6)	
H3AN	0.115 (4)	0.073 (3)	0.800 (3)	0.076 (9)*	
H3BN	0.062 (3)	−0.002 (3)	0.896 (2)	0.074 (8)*	
C2	0.3685 (3)	0.6714 (3)	0.68520 (18)	0.0499 (5)	
H2	0.467722	0.610398	0.689533	0.060*	
C3	0.2332 (3)	0.6009 (3)	0.74971 (17)	0.0493 (5)	
H3A	0.281782	0.610110	0.826354	0.059*	
H3B	0.134816	0.661355	0.748204	0.059*	
C4	0.1747 (3)	0.4250 (2)	0.69944 (17)	0.0450 (4)	
C5	0.1826 (3)	0.3784 (3)	0.58006 (16)	0.0474 (5)	
C6	0.1305 (3)	0.2204 (3)	0.52088 (19)	0.0606 (6)	
H6	0.091678	0.139229	0.558488	0.073*	
C7	0.1353 (3)	0.1822 (3)	0.4087 (2)	0.0681 (7)	
H7	0.104231	0.075412	0.371456	0.082*	
C8	0.1866 (3)	0.3031 (4)	0.35112 (19)	0.0650 (7)	
H8	0.186752	0.278143	0.274751	0.078*	
C9	0.2373 (3)	0.4599 (3)	0.40644 (19)	0.0607 (6)	
H9	0.269457	0.541354	0.367367	0.073*	
C10	0.2406 (3)	0.4967 (3)	0.52061 (17)	0.0500 (5)	
C11	0.4330 (3)	0.8494 (3)	0.72737 (18)	0.0506 (5)	
C12	0.4111 (3)	0.9656 (3)	0.6620 (2)	0.0604 (6)	
H12	0.352887	0.934776	0.590588	0.073*	
C13	0.4752 (3)	1.1273 (3)	0.7020 (2)	0.0668 (7)	
H13	0.460876	1.204685	0.657535	0.080*	
C14	0.5595 (3)	1.1727 (3)	0.8070 (2)	0.0624 (6)	
C15	0.5862 (4)	1.0597 (3)	0.8729 (2)	0.0719 (7)	
H15	0.645873	1.091518	0.943806	0.086*	
C16	0.5233 (4)	0.8983 (3)	0.8325 (2)	0.0682 (7)	
H16	0.541890	0.821123	0.876540	0.082*	
C17	0.0759 (3)	0.2312 (2)	0.91799 (17)	0.0470 (5)	

### Atomic displacement parameters (Å^2^)

**Table:**

	*U*^11^	*U*^22^	*U*^33^	*U*^12^	*U*^13^	*U*^23^
S1	0.0973 (4)	0.0372 (3)	0.0360 (3)	0.0047 (3)	0.0169 (3)	0.0114 (2)
Cl1	0.1088 (6)	0.0550 (4)	0.1009 (6)	−0.0103 (4)	−0.0092 (4)	0.0184 (4)
O1	0.0846 (11)	0.0556 (9)	0.0446 (8)	0.0059 (8)	0.0172 (7)	0.0219 (7)
N1	0.0673 (11)	0.0448 (9)	0.0385 (9)	0.0057 (8)	0.0134 (8)	0.0132 (7)
N2	0.0761 (12)	0.0362 (9)	0.0386 (9)	0.0049 (8)	0.0174 (8)	0.0121 (7)
N3	0.127 (2)	0.0374 (10)	0.0482 (12)	0.0088 (11)	0.0328 (12)	0.0121 (9)
C2	0.0547 (11)	0.0521 (12)	0.0491 (12)	0.0100 (9)	0.0147 (9)	0.0200 (9)
C3	0.0593 (12)	0.0485 (11)	0.0438 (11)	0.0050 (9)	0.0130 (9)	0.0163 (9)
C4	0.0498 (11)	0.0480 (11)	0.0406 (10)	0.0083 (8)	0.0092 (8)	0.0143 (8)
C5	0.0500 (11)	0.0577 (12)	0.0380 (10)	0.0090 (9)	0.0085 (8)	0.0154 (9)
C6	0.0730 (15)	0.0630 (14)	0.0427 (12)	−0.0017 (11)	0.0043 (10)	0.0101 (10)
C7	0.0738 (16)	0.0771 (17)	0.0467 (13)	0.0005 (13)	0.0025 (11)	0.0017 (12)
C8	0.0578 (13)	0.101 (2)	0.0356 (11)	0.0103 (13)	0.0068 (9)	0.0095 (12)
C9	0.0627 (13)	0.0833 (17)	0.0434 (12)	0.0128 (12)	0.0161 (10)	0.0244 (11)
C10	0.0519 (11)	0.0594 (13)	0.0440 (11)	0.0128 (9)	0.0121 (9)	0.0173 (9)
C11	0.0518 (11)	0.0508 (12)	0.0546 (12)	0.0066 (9)	0.0154 (9)	0.0198 (10)
C12	0.0671 (14)	0.0556 (13)	0.0624 (14)	0.0069 (10)	0.0047 (11)	0.0241 (11)
C13	0.0729 (15)	0.0549 (14)	0.0773 (17)	0.0061 (11)	0.0021 (13)	0.0305 (12)
C14	0.0617 (13)	0.0518 (13)	0.0755 (16)	0.0005 (10)	0.0090 (12)	0.0208 (11)
C15	0.0828 (17)	0.0661 (16)	0.0636 (15)	−0.0061 (13)	−0.0029 (13)	0.0199 (12)
C16	0.0828 (17)	0.0581 (14)	0.0663 (16)	0.0004 (12)	0.0026 (13)	0.0283 (12)
C17	0.0642 (12)	0.0382 (10)	0.0407 (10)	0.0027 (9)	0.0109 (9)	0.0129 (8)

### Geometric parameters (Å, º)

**Table:**

S1—C17	1.687 (2)	C5—C6	1.398 (3)
Cl1—C14	1.746 (2)	C6—C7	1.372 (3)
O1—C10	1.361 (3)	C6—H6	0.9300
O1—C2	1.433 (3)	C7—C8	1.383 (4)
N1—C4	1.281 (3)	C7—H7	0.9300
N1—N2	1.375 (2)	C8—C9	1.373 (4)
N2—C17	1.351 (3)	C8—H8	0.9300
N2—H2N	0.85 (3)	C9—C10	1.387 (3)
N3—C17	1.315 (3)	C9—H9	0.9300
N3—H3AN	0.85 (3)	C11—C16	1.385 (3)
N3—H3BN	0.88 (3)	C11—C12	1.385 (3)
C2—C11	1.511 (3)	C12—C13	1.383 (3)
C2—C3	1.514 (3)	C12—H12	0.9300
C2—H2	0.9800	C13—C14	1.364 (4)
C3—C4	1.505 (3)	C13—H13	0.9300
C3—H3A	0.9700	C14—C15	1.374 (4)
C3—H3B	0.9700	C15—C16	1.381 (4)
C4—C5	1.468 (3)	C15—H15	0.9300
C5—C10	1.396 (3)	C16—H16	0.9300
			
C10—O1—C2	114.56 (16)	C8—C7—H7	120.1
C4—N1—N2	118.41 (17)	C9—C8—C7	120.2 (2)
C17—N2—N1	117.54 (17)	C9—C8—H8	119.9
C17—N2—H2N	117.8 (17)	C7—C8—H8	119.9
N1—N2—H2N	122.4 (17)	C8—C9—C10	120.0 (2)
C17—N3—H3AN	117 (2)	C8—C9—H9	120.0
C17—N3—H3BN	121.9 (18)	C10—C9—H9	120.0
H3AN—N3—H3BN	121 (3)	O1—C10—C9	117.1 (2)
O1—C2—C11	107.89 (17)	O1—C10—C5	121.98 (19)
O1—C2—C3	110.01 (18)	C9—C10—C5	120.9 (2)
C11—C2—C3	114.02 (18)	C16—C11—C12	118.5 (2)
O1—C2—H2	108.3	C16—C11—C2	119.6 (2)
C11—C2—H2	108.3	C12—C11—C2	121.8 (2)
C3—C2—H2	108.3	C13—C12—C11	120.5 (2)
C4—C3—C2	109.75 (17)	C13—C12—H12	119.7
C4—C3—H3A	109.7	C11—C12—H12	119.7
C2—C3—H3A	109.7	C14—C13—C12	119.7 (2)
C4—C3—H3B	109.7	C14—C13—H13	120.1
C2—C3—H3B	109.7	C12—C13—H13	120.1
H3A—C3—H3B	108.2	C13—C14—C15	121.0 (2)
N1—C4—C5	117.19 (19)	C13—C14—Cl1	119.26 (19)
N1—C4—C3	126.53 (19)	C15—C14—Cl1	119.7 (2)
C5—C4—C3	116.28 (17)	C14—C15—C16	119.1 (3)
C10—C5—C6	117.4 (2)	C14—C15—H15	120.4
C10—C5—C4	119.36 (19)	C16—C15—H15	120.4
C6—C5—C4	123.21 (19)	C15—C16—C11	121.0 (2)
C7—C6—C5	121.6 (2)	C15—C16—H16	119.5
C7—C6—H6	119.2	C11—C16—H16	119.5
C5—C6—H6	119.2	N3—C17—N2	117.00 (19)
C6—C7—C8	119.7 (2)	N3—C17—S1	122.99 (17)
C6—C7—H7	120.1	N2—C17—S1	120.00 (16)
			
C4—N1—N2—C17	−169.8 (2)	C8—C9—C10—C5	−4.0 (3)
C10—O1—C2—C11	178.22 (17)	C6—C5—C10—O1	−177.7 (2)
C10—O1—C2—C3	−56.8 (2)	C4—C5—C10—O1	3.8 (3)
O1—C2—C3—C4	57.2 (2)	C6—C5—C10—C9	3.4 (3)
C11—C2—C3—C4	178.59 (17)	C4—C5—C10—C9	−175.0 (2)
N2—N1—C4—C5	−178.00 (17)	O1—C2—C11—C16	−173.0 (2)
N2—N1—C4—C3	2.7 (3)	C3—C2—C11—C16	64.5 (3)
C2—C3—C4—N1	150.2 (2)	O1—C2—C11—C12	4.2 (3)
C2—C3—C4—C5	−29.1 (2)	C3—C2—C11—C12	−118.3 (2)
N1—C4—C5—C10	179.93 (19)	C16—C11—C12—C13	−1.3 (4)
C3—C4—C5—C10	−0.7 (3)	C2—C11—C12—C13	−178.5 (2)
N1—C4—C5—C6	1.6 (3)	C11—C12—C13—C14	−0.5 (4)
C3—C4—C5—C6	−179.1 (2)	C12—C13—C14—C15	1.8 (4)
C10—C5—C6—C7	−0.2 (4)	C12—C13—C14—Cl1	−178.7 (2)
C4—C5—C6—C7	178.2 (2)	C13—C14—C15—C16	−1.3 (4)
C5—C6—C7—C8	−2.4 (4)	Cl1—C14—C15—C16	179.2 (2)
C6—C7—C8—C9	1.9 (4)	C14—C15—C16—C11	−0.5 (4)
C7—C8—C9—C10	1.3 (4)	C12—C11—C16—C15	1.8 (4)
C2—O1—C10—C9	−155.12 (19)	C2—C11—C16—C15	179.1 (2)
C2—O1—C10—C5	26.0 (3)	N1—N2—C17—N3	9.8 (3)
C8—C9—C10—O1	177.1 (2)	N1—N2—C17—S1	−171.68 (15)

### Hydrogen-bond geometry (Å, º)

**Table:**

*D*—H···*A*	*D*—H	H···*A*	*D*···*A*	*D*—H···*A*
N2—H2*N*···S1^i^	0.85 (3)	2.65 (3)	3.480 (2)	167 (2)
N3—H3*BN*···S1^ii^	0.88 (3)	2.52 (3)	3.392 (2)	171 (2)

Symmetry codes: (i) −*x*, −*y*+1, −*z*+2; (ii) −*x*, −*y*, −*z*+2.
